# Evaluation of radiotherapy combined with targeted therapy and concurrent radiotherapy, chemotherapy in the treatment of Non-Small Cell Lung Cancer with brain metastasis

**DOI:** 10.12669/pjms.36.3.1626

**Published:** 2020

**Authors:** Yanfeng Sun, Xiaohui Guo, Lingling Zhang, Wenqian Zhang, Yuqin Zuo

**Affiliations:** 1 Yanfeng Sun, Department of Oncology, Binzhou People’s Hospital, Shandong 256610, China; 2 Xiaohui Guo, Department of Pediatric, Binzhou People’s Hospital, Shandong 256610, China; 3 Lingling Zhang, Department of Oncology, Binzhou People’s Hospital, Shandong 256610, China; 4 Wenqian Zhang, Department of Orthopedics, Binzhou People’s Hospital, Shandong 256610, China; 5 Yuqin Zuo, Department of Endoscopic, Binzhou People’s Hospital, Shandong 256610, China

**Keywords:** Brain radiotherapy, targeted therapy, non-small cell lung cancer with brain metastasis, disease control rate

## Abstract

**Objective:**

To compare and analyze the clinical efficacy of brain radiotherapy combined with targeted therapy and concurrent radiotherapy and chemotherapy in the treatment of non-small cell lung cancer (NSCLC) with brain metastasis.

**Methods:**

Fifty-eight patients with NSCLC with brain metastasis who were admitted to our hospital between October 2016 and October 2017 were randomly divided into a control group and an observation group, 29 cases in each group. The control group was treated with concurrent radiotherapy and chemotherapy, while the observation group was treated with whole brain radiotherapy plus targeted therapy. The disease control rate, adverse reactions and survival condition were compared between the two groups.

**Results:**

The disease control rate of the observation group was 68.97%, significantly higher than 41.38% of the control group (P<0.05); the total incidence of adverse reactions in the observation group was 6.90%, significantly lower than 24.14% of the control group (P<0.05); the median survival time of the observation group was (16.81±5.32) months, significantly longer than that of the control group ((9.76±3.25) months). The one-year and two-year survival rates in the observation group were significantly higher than those in the control group (P<0.05).

**Conclusion:**

Whole brain radiotherapy combined with targeted therapy is superior to concurrent radiotherapy and chemotherapy in the treatment of NSCLC with brain metastasis and has high safety. It can effectively prolong the life span of patients and is worth clinical promotion and application.

## INTRODUCTION

Lung cancer is the most common malignant tumor in our clinical practice. Nearly 80% of lung cancer patients suffer from non-small cell lung cancer (NSCLC), and about 25% of NSCLC is prone to brain metastasis during the course of disease.^1^,^2^ The mortality and morbidity of NSCLC are high. Some studies reported that the mortality and morbidity of NSCLC accounted for about 80% of the total lung cancer.^3^ At present, the treatment of NSCLC with brain metastasis includes whole brain radiotherapy, surgical resection, stereotactic therapy, chemotherapy and targeted therapy.^4^,^5^ A study has shown that the median overall survival time of untreated NSCLC patients with brain metastases is less than 3 to 6 months.^6^ Chemotherapy is an important means to treat NSCLC. Past studies have shown that chemotherapeutics do not cross the blood-brain barrier; however, recent studies have shown that platinum combined with cytotoxic drugs can benefit patients.^7^ With the advances in research, targeted therapy has increasingly become one of the important means to treat NSCLC with brain metastasis.^8^ Epidermal growth factor receptor tyrosine kinase inhibitor (EGFR-TKI) is currently widely used in the treatment of NSCLC. A large number of clinical studies have shown that EGFR-TKI can significantly prolong the disease-free survival of patients compared with chemotherapy.^9^,^10^ At present, the treatment of cancer patients tends to be multidisciplinary, but there are still many controversies about the best treatment for NSCLC with brain metastasis. The aim of this study was to compare the effects of combination of whole brain radiotherapy plus targeted therapy and concurrent radiotherapy and chemotherapy in the treatment of NSCLC with brain metastasis in order to provide an evidence-based basis for the selection of clinical treatment.

## METHODS

### General data:

Fifty-eight patients with NSCLC with brain metastasis who were admitted to our hospital between October 2016 and October 2017 were selected as the research subjects. The patients were divided into a control group and an observation group by digital random grouping method, with 29 cases each group. In the observation group, there were 17 males and 12 females, aged from 32 to 75 years, with an average age of (46.57±4.11) years. As to pathological types, there were 8 cases of adenocarcinoma, 6 cases of squamous cell carcinoma, 5 cases of adenosquamous carcinoma, 6 cases of large cell carcinoma while the type of carcinoma was not known in four cases. There were 16 males and 14 females in the control group, aged 33 to 76 years, with an average age of (47.25±4.68) years. As to pathological types, there were 9 cases of adenocarcinoma, 5 cases of squamous cell carcinoma, 5 cases of adenosquamous carcinoma, 5 cases of large cell carcinoma and five cases of unknown pathology. The results could be compared as there was no significant difference in general data between the two groups (P>0.05). This study was approved by the ethics committee of our hospital. All the selected subjects had informed consent. (Dated: 9 September 2019)

### Conventional nursing intervention

Control group received conventional nursing intervention. Charge nurse informed patients with cause of lung cancer, treatment method, treatment effect, prognosis, 7 matters needing attention before and after surgery such as diet, rest, knowledge relating to management of pain on wound and various exercise methods. One day before surgery, operating room nurse informed patients with operating time, anesthesia method, preparation before surgery, pain and bleeding that may occur after surgery and matters needing attention after surgery.

### Inclusion and exclusion criteria

Inclusion criteria included all patients who met the diagnostic criteria of brain metastasis of NSCLC according to pathological examination and computed tomography (CT) or Magnetic Resonance Imaging (MRI) examination;^11^ the estimated survival time was more than 3 months; there was no history of radiotherapy and chemotherapy in the past. Exclusive criteria were: patients with severe cardiopulmonary, liver and kidney insufficiency; allergy to drugs involved in this study; patients with severe mental disorders.

### Methods

The control group received concurrent radiotherapy and chemotherapy. The whole brain was irradiated with 8 m V X-ray for 5 times per week, 3 Gy per time, totaling 30 Gy. The treatment lasted for 2 weeks. On the first day of chemotherapy, paclitaxel and pemetrexed were intravenously infused at the dose of 175 mg/m^2^ and 500 mg/m^2^ respectively; cisplatin was infused intravenously at the dose of 25 mg/m^2^ from the first day to the third day. There was an interval of 3 weeks or 4 weeks between every course, totaling 3 courses.

The observation group received brain radiotherapy combined with targeted therapy. Firstly, the whole brain of the patient was horizontally treated with 8 m V X-ray, 2 Gy/time, 5 w/time, 40 Gy for 4 weeks. Moreover, the patient orally took gefitinib (Astra Zeneca UK Ltd., 40090529, 0.25 g) for targeted therapy, 250 mg per day; for patients diagnosed as squamous cell cancer, erlotinib (Schwarz Pharma Manufacturing, Inc., H20090225, tabella), 150 mg per day. Targeted therapy stopped two months after the completion of radiotherapy.

During the treatment, the two groups cooperated with the following nursing: (1) nursing of radiation brain edema: radiotherapy will aggravate the brain edema of brain metastasis patients and induce symptoms such as dizziness, nausea and disturbance of consciousness. Thus the patients needed to actively cooperate with the treatment and were forbidden to eat spicy and irritating food; (2) psychological nursing: The confidence of patients on treatment was strengthened to alleviate patients’ fear and resistance and promote patients to actively cooperate with the treatment to ensure the smooth implementation of radiotherapy and chemotherapy; (3) nursing of digestive tract reaction: during the course of radiotherapy and chemotherapy, many adverse reaction symptoms of digestive tract occurred, thus antiemetic agent was given to patients 30 minutes after temozolomide chemotherapy to alleviate the adverse reaction of patients; help was given in time; the vomiting reaction and the amount of vomit in and out were closely observed, and the dehydration status was evaluated.

### Observation indicators and evaluation criteria

The clinical efficacy of the two groups was evaluated by World Health Organization (WHO) standard;^12^ the increase of tumor volume exceeding 25% or new lesions appearing was evaluated as progressive disease (PD); the shrinking of tumor volume less than 50% or increase less than 25% was evaluated as SD (stable disease); no recurrence in four weeks or the shrinking of tumor volume no less than 50% was evaluated as PR (partial remission); disappearance of tumors, no recurrence in 4 weeks and no appearance of new lesions was evaluated as CR (complete remission); disease control rate (DCR)=(number of cases of SD+number of cases of PR+number of cases of CR)/total number of cases*100%. The total incidence of adverse reactions (diarrhea, impaired liver function, radiation intracranial hypertension) was compared between the two groups. The two groups were followed up for 2 years, and the one-year and two-year survival rates and median survival time were also compared between the two groups.

### Statistical Analysis

SPSS20.0 statistical software was used for processing the data. Measurement data are expressed as Mean±Standard Deviation. t test was used. Counting data was expressed as rate (%), and X^2^ test was used. P<0.05 indicated that there was significant difference.

## RESULTS

### Comparison of disease control rate between two groups

The disease control rate of the observation group was 68.97%, which was significantly higher than that of the control group (41.38%, P<0.05, Table-I).

**Table-I:** Disease control rate between two groups (%)

Group	PD	SD	PR	CR	DCR
Observation group	9(31.03)	7(24.14)	8(27.59)	5(17.24)	20(68.97)
Control group	17(58.62)	4(13.79)	5(17.24)	3(10.34)	12(41.38)
X^2^	/	/	/	/	7.841
P	/	/	/	/	<0.05

### Comparison of the total incidence of adverse reactions between the two groups

The total incidence of adverse reactions in the observation group was 6.90%, significantly lower than that in the control group (24.14%, P<0.05, Table-II).

**Table-II:** Total incidence of adverse reactions between the two groups (%).

Group	Diarrhea	Impaired liver function	Radioactive intracranial hypertension	Total incidence of adverse reactions
Observation group	1(3.45)	1(3.45)	0(0)	2(6.90)
Control group	4(13.79)	2(6.90)	1(3.45)	7(24.14)
X^2^	/	/	/	4.286
P	/	/	/	<0.05

### Comparison of the median survival time between the two groups

The median survival time of the observation group was 16.81±5.32 months, significantly longer than that of the control group (9.76±3.25 months), and the difference was statistically significant (t=6.3229, P<0.05); the one-year and two-year survival rates of the observation group were significantly higher than those of the control group (P<0.05, Table-III).

**Table-III:** Median survival time between two groups.

Group	One-year survival rate	Two-year survival rate
Observation group	22(75.86)	12(41.38)
Control group	12(41.38)	5(17.24)
X^2^	8.112	5.014
P	<0.05	<0.05

## DISCUSSION

The incidence of NSCLC with brain metastasis is about 20%-40%, and 10%-25% of NSCLC patients have brain metastasis at the first diagnosis. The median survival time of untreated patients with brain metastasis is only 3 months.^13^,^14^ This high rate of metastasis need more effective treatments. At present, concurrent radiotherapy and chemotherapy is often used in the treatment of malignant tumors, but a large number of studies have shown that the effect of chemotherapy is not good and the adverse reactions are large although concurrent radiotherapy and chemotherapy can prolong the survival time of patients,^15^ which has a serious impact on the quality of life of patients.

In recent years, the treatment of cancer is becoming more and more precise, and targeted therapy has become increasingly important. EGFR is the most common driving gene of NSCLC, and the first and second generation EGFR-TKIs have become the first-line treatment for advanced NSCLC patients with positive EGFR mutation because of their better efficacy and higher survival benefits.^16^,^17^

In this study, gefitinib and erlotinib were selected for targeted therapy; the former belongs to epidermal growth factor receptor (EGFR) tyrosine kinase inhibitors, and the latter belongs to quinazoline derivatives; both of them are small molecular compounds and could inhibit the metastasis and growth of tumors by inhibiting the activity of EGFR-TKI, thus causing apoptosis of tumour cells.^18^,^19^ Relevant studies have shown that the change of EGFR expression level is closely related to the progression and spread of brain metastasis, the blood-brain barrier will be damaged once brain metastasis occurs, targeted therapy at that time will make drugs more easily enter the blood-brain barrier, and the combination with radiotherapy can increase the permeability of blood-brain barrier to the greatest extent.^20^,^21^ Jiang et al. has found that radiotherapy (whole brain) combined with targeted therapy for NSCLC with brain metastasis has a significant effect,^22^ the survival rate of patients after 6 months of treatment was 95.45%, and the one-year survival rate was 68.18%, i.e., it could effectively prolong the survival of patients. The results showed that the control rate of brain metastasis in the observation group was significantly higher than that in the control group, suggesting that the effect of radiotherapy (whole brain) combined with targeted therapy was more significant in the treatment of NSCLC with brain metastasis and could effectively improve the quality of life; the median survival time of the observation group was better than that of the control group, suggesting that radiotherapy (whole brain) combined with targeted therapy could effectively improve the quality of life of patients with NSCLC and prolong survival time, which was consistent with the results of Li et al.^23^ In addition, the study also showed that the incidence of adverse reactions in the observation group was significantly lower than that in the control group, suggesting that the combination of radiotherapy (whole brain) and targeted therapy was safe. The reason given may be that the targeted therapy with strong targeting could accurately combine with the target site and promote the death of cancer cell, without affecting the surrounding cells and organs of cancer tissue.

## CONCLUSION

In conclusion, for NSCLC patients with brain metastasis, brain radiotherapy combined with targeted therapy can not only improve the disease control rate and reduce adverse reactions, but also effectively prolong the survival time of patients and improve survival rate. However, due to the small number of patients selected, the long-term quality of life of the two groups has not been investigated in this study, which needs further investigation.

### Authors’ Contribution:

**YFS & XHG:** Study design, data collection and analysis.

**YFS, LLZ & WQZ:** Manuscript preparation, drafting and revising.

**YFS & YQZ:** Review and final approval of manuscript.

## References

[1] Cooper WA, Tran T, Vilain RE, Madore J, Selinger CI, Kohonen-Corish M (2015). PD-L1 expression is a favorable prognostic factor in early stage non-small cell carcinoma. Lung Cancer.

[2] Langer CJ, Mehta MP (2005). Current management of brain metastases, with a focus on systemic options. J Clin Oncol.

[3] Jaeger S, Lee BH, Mosher R (2015). Evaluation of PD-L1 mRNA and protein expression in non-small cell lung and hepatocellular carcinoma. Cancer Res.

[4] Xie SS, Tan M, Lin HY, Xu L, Shen CX, Yuan Q (2015). Overexpression of adenylate cyclase-associated protein 1 may predict brain metastasis in non-small cell lung cancer. Oncol Rep.

[5] Preusser M, Winkler F, Valiente M, Manegold C, Moyal E, Widhalm G (2018). Recent advances in the biology and treatment of brain metastases of non-small cell lung cancer:summary of a multidisciplinary roundtable discussion. ESMO Open.

[6] Counago F, Rodrguez A, Calvo P, Luna J, Monroy JL, Taboada B (2017). Targeted therapy combined with radiotherapy in non-small-cell lung cancer:a review of the Oncologic Group for the Study of Lung Cancer (Spanish Radiation Oncology Society). Clin Transl Oncol.

[7] Wallerek S, Sorensen JB (2015). Biomarkers for efficacy of adjuvant chemotherapy following complete resection in NSCLC stages I-IIIA. Eur Respir Rev.

[8] Baek MY, Ahn HK, Park KR, Park HS, Kang SM, Park I (2018). Epidermal growth factor receptor mutation and pattern of brain metastasis in patients with non-small cell lung cancer. Korean J Intern Med.

[9] Baik CS, Chamberlain MC, Chow LQ (2015). Targeted therapy for brain metastases in EGFR-mutated and ALK-rearranged non-small-cell lung cancer. J Thorac Oncol.

[10] Khalifa J, Amini A, Popat S, Gaspar LE, Faivre-Finn C (2016). Brain metastases from NSCLC:radiation therapy in the era of targeted therapies. J Thorac Oncol.

[11] Qin H, Zhang KQ, Li WH, Hao LJ, Ruan ZH (2015). Combining whole brain radiotherapy with target drug for non-small cell lung cancer with multiple brain metastases:a systematic review. Chin J Cancer Prev Treat.

[12] Zhang XX, Bi DP (2015). Observation of clinical effect of radiotherapy combined with chemotherapy for non-small cell lung cancer with brain metastasis. J Clin Pulmon Med.

[13] Sperduto PW, Kased N, Roberge D, Xu Z, Shanley R, Luo X (2012). Summary report on the graded prognostic assessment:An accurate and facile diagnosis-specific tool to estimate survival for patients with brain metastases. J Clin Oncol.

[14] Jeene PM, de Vries KC, van Nes JGH, Kwakman JJM, Wester G, Rozema T (2018). Survival after whole brain radiotherapy for brain metastases from lung cancer and breast cancer is poor in 6325 Dutch patients treated between 2000 and 2014. Acta Oncol.

[15] Guckenberger M (2015). Stereotactic body Radiothera-py in operable patients with stage I NSCLC:where is the evidence?. Expert Rev Anticancer Ther.

[16] Rosell R, Carcereny E, Gervais R, Vergnenegre A, Massuti B, Felip E (2012). Erlotinib versus standard chemotherapy as first-line treatment for European patients with advanced EGFR mutation-positive non-small-cell lung cancer (EURTAC):A multicentre, open-label, randomised phase 3 trial. Lancet Oncol.

[17] Jiang X, Wang W, Zhang Y (2016). Clinical analysis of icotinib on beneficiary of advanced non-small cell lung cancer with EGFR common mutation. Zhongguo Fei Ai Za Zhi.

[18] Wang JL, Xie CH (2016). Whole brain radiotherapy combined with chemotherapy and gefitinib targeted therapy in the treatment of advanced non-small cell lung cancer with brain metastasis. Chin J Pract Med.

[19] Shi Y, Ma JB, Wang Y, Chen XJ, Gai L, Yao NH (2017). Clinical efficacy of whole brain radiotherapy combined with targeted therapy in treating lung adenocarcinoma with brain metastases. Jiangsu Med J.

[20] Pesce GA, Klingbiel D, Ribi K, Zouhair A, von Moos R, Schlaeppi M (2012). Outcome, quality of life and cognitive function of patients with brain metastases from non-small cell lung cancer treated with whole brain radiotherapy combined with gefitinib or temozolomide. A randomised phase II trial of the Swiss Group for Clinical Cancer Research (SAKK 70/03). Eur J Cancer.

[21] Tao LM, Le W, Wang F, Wan BL (2017). Clinical effects of general chemotherapy combined with DC-CIK cell in the treatment of non small cell lung cancer. China Mod Med.

[22] Jiang YL, Chen HL, Shen G (2015). Curative effect observation of temozolomide combined with whole brain radiotherapy in the brain metastases patients of non-small cell lung cancer. Mod Med J.

[23] Li W, Zhang HY, Song TT, Hu FJ (2015). Efficacy and safety of combination of radiotherapy and chemotherapy in treatment drug therapy of patients with tar-get of brain metastases origin from lung cancer. J Difficult Complicated Cases.

